# Milk antibody response after 3^rd^ COVID-19 vaccine and SARS-CoV-2 infection and implications for infant protection

**DOI:** 10.1016/j.isci.2023.107767

**Published:** 2023-08-29

**Authors:** Yarden Golan, Mikias Ilala, Lin Li, Caryl Gay, Soumya Hunagund, Christine Y. Lin, Arianna G. Cassidy, Unurzul Jigmeddagva, Yusuke Matsui, Nida Ozarslan, Ifeyinwa V. Asiodu, Nadav Ahituv, Valerie J. Flaherman, Stephanie L. Gaw, Mary Prahl

**Affiliations:** 1 Department of Bioengineering and Therapeutic Sciences, University of California, San Francisco, and Institute for Human Genetics, University of California, San Francisco, San Francisco, CA, USA; 2 Department of Pediatrics, University of California, San Francisco, San Francisco, CA, USA; 3 Division of Experimental Medicine, Department of Medicine, University of California, San Francisco, San Francisco, CA, USA; 4 Division of Maternal-Fetal Medicine, Department of Obstetrics, Gynecology, and Reproductive Sciences, University of California San Francisco, San Francisco, CA, USA; 5 Center for Reproductive Sciences, Department of Obstetrics, Gynecology, and Reproductive Sciences, University of California San Francisco, San Francisco, CA, USA; 6 Department of Family Health Care Nursing, University of California, San Francisco, San Francisco, CA, USA; 7 Gladstone Institute of Virology, Gladstone Institutes, San Francisco, CA, USA; 8 Michael Hulton Center for HIV Cure Research at Gladstone, San Francisco, CA, USA; 9 Division of Pediatric Infectious Diseases and Global Health, University of California, San Francisco, San Francisco, CA, USA

**Keywords:** Health sciences, Pediatrics, Immunology, Virology

## Abstract

Little is known about the persistence of human milk anti-SARS-CoV-2 antibodies after 2^nd^ and 3^rd^ vaccine doses and infection following 3^rd^ dose. In this study, human milk, saliva, and blood samples were collected from 33 lactating individuals before and after vaccination and infection. Antibody levels were measured using ELISA and symptoms were assessed using questionnaires. We found that after vaccination, milk anti-SARS-CoV-2 antibodies persisted for up to 8 months. In addition, distinct patterns of human milk IgA and IgG production and higher milk RBD-blocking activity was observed after infection compared to 3-dose vaccination. Infected mothers reported more symptoms than vaccinated mothers. We examined the persistence of milk antibodies in infant saliva after breastfeeding and found that IgA was more abundant compared to IgG. Our results emphasize the importance of improving the secretion of IgA antibodies to human milk after vaccination to improve the protection of breastfeeding infants.

## Highlights

• Milk anti-SARS-CoV-2 antibodies persist 6–8 months following vaccination
• Distinct patterns of milk IgA and IgG are produced after infection versus vaccination
• Infant saliva IgA is more abundant and persists compared to IgG after breastfeeding

## Graphical abstract

**Figure:**
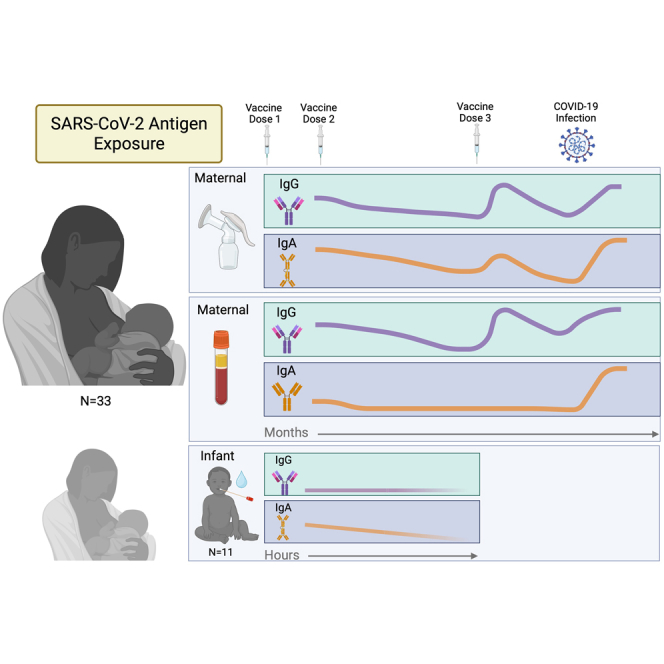

## Introduction

Exclusive breastfeeding is recommended for infants up to 6 months of age and is recommended by the American Academy of Pediatrics to be continued with the introduction of complementary foods to the infant diet for 2 years of age or longer.^1^ Breastfeeding provides short and long-term protective effects from a number of diseases^1^ and breastfeeding duration and exclusivity is specifically associated with reduced risk of lower respiratory tract infections in infants.^2^ Human milk contains multiple factors that provide anti-viral protection to the infant including immune cells, extracellular vesicles, cytokines, enzymes, and antibodies.^3^^,^^4^^,^^5^ The breast is a unique organ in that despite not having a direct mucosal surface, it provides passive mucosal immunity including IgA, IgM, and IgG to the breastfeeding infant. IgA, the predominant human milk antibody, is typically present in its secretory form (sIgA) and provides passive mucosal defense for the infant’s respiratory and digestive systems.^5^^,^^6^^,^^7^ In contrast, IgG, despite being the most prominent antibody in blood, is present in its monomeric form in human milk at lower levels than IgA or IgM, yet helps provide protection against enteric pathogens.^8^^,^^9^ Numerous studies have shown the presence of anti-SARS-CoV-2 antibodies in human milk after two doses of mRNA-based COVID-19 vaccines.^10^^,^^11^^,^^12^^,^^13^^,^^14^^,^^15^^,^^16^^,^^17^^,^^18^^,^^19^^,^^20^^,^^21^ Specifically, IgA and IgG against the spike (S) protein of SARS-CoV-2 have been found in human milk after both vaccination and infection.^7^ However, differential antibody dynamics based on the type of preceding antigen exposure—vaccination versus infection—has been described. Milk IgG increases significantly after the 2^nd^ vaccine dose, while secretory IgA significantly rises after SARS-CoV-2 infection with minimal increase of IgG.^16^^,^^18^ As the COVID-19 pandemic and vaccine strategies have evolved over time, further information is needed on the potency and duration of the antibody response in milk beyond the 2^nd^ vaccine dose and the impact of hybrid immunity from infections that have become increasingly common in the Omicron era.

Young infants are at increased risk of severe disease and hospitalization from COVID-19 as compared to older children.^22^ Current COVID-19 vaccinations are not approved until infants reach at least 6 months of age. Vaccination during pregnancy may provide some protection to the infant, as infants that were born to fully vaccinated mothers have a lower risk for SARS-CoV-2 infection^23^ and hospitalization^24^ compared to unvaccinated mothers. However, due to the lack of inclusion of lactating individuals in COVID-19 vaccination clinical trials, there is limited data on symptomatology and immune protection following vaccination and infection in lactating individuals and breastfeeding infants. Further information is needed on immune protection against SARS-CoV-2 during the vulnerable first months of infancy including the persistence of anti-SARS-CoV-2 antibodies in milk after vaccination and level of antibody transfer to the infant.

Here, we present longitudinal assessment of anti-SARS-CoV-2 milk antibody levels of lactating individuals after 2- or 3-dose vaccine series, as well as following infection occurring after 3^rd^ vaccine dose. We assessed maternal and infant symptomatology after vaccination or infection. Lastly, we assessed the presence and duration of passively transferred antibodies in the saliva of breastfeeding infants.

## Results

### Participant cohort

**Figure 1:**
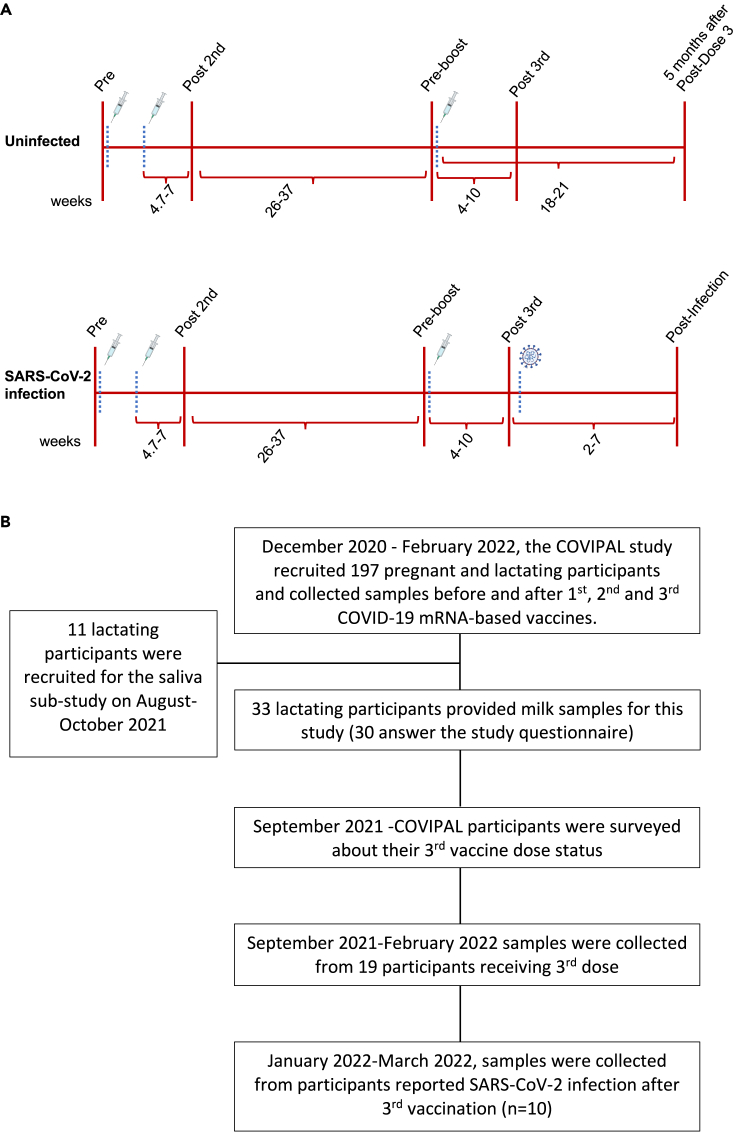
COVIPAL (A) Study timeline. (B) Enrollment schema.

Human milk samples were collected from 33 lactating individuals that received the first 2 doses of an mRNA-based COVID-19 vaccine (BTN162b2 or mRNA-1273) during pregnancy (n = 25) or lactation (n = 8) (Table 1). Figure 1 describes the timing of samples collection and recruitment strategy for this study. Twenty-six individuals from this cohort received the 3^rd^ dose of COVID-19 vaccine and reported their symptoms after vaccination (Table 2). Out of the 26 participants receiving 3^rd^ dose, 19 participants (3^rd^ dose subgroup) provided samples for antibodies assessment after 3^rd^ dose and their clinical characteristics are shown in Table 3. Of these 19 participants that received a 3^rd^ dose, 10 experienced SARS-CoV-2 infection from December 2021-March 2022, during the Omicron wave (SARS-CoV-2 B.1.1.529) in the San Francisco Bay Area (Table 3). Additional fourteen participants provided milk and/or saliva and infant saliva samples (after 2^nd^ or 3^rd^ dose).

**Table 1:** Sample characteristics, overall and for the 3^rd^ dose subgroups

Demographic and primary vaccine characteristics	Total sample (n=30^a^)	3^rd^ dose subgroups	
		Uninfected (n=9)	SARS-CoV-2 infection (n=10)
**Demographic Characteristics**			
Maternal age, years			
Mean (SD)	37.2 (4.1)	37.8 (3.3)	36.3 (4.0)
Median (min, max)	37.2 (29.3, 44.7)	38.0 (31.4, 41.6)	37.3 (29.3, 42.0)
Race/ethnicity, % (n)			
Asian	20% (6)	22% (2)	10% (1)
Black or African American	3% (1)		10% (1)
Hispanic/Latina	3% (1)		
White/Caucasian	70% (21)	67% (6)	80% (8)
More than 1 race/ethnicity	3% (1)	11% (1)	
Education, % (n)			
Some college	3% (1)	11% (1)	
College graduate	23% (7)	22% (2)	40% (4)
Advanced degree	73% (22)	67% (6)	60% (6)
Employed in health care, % (n)			
Yes, providing direct patient care	40% (12)	67% (6)	30% (3)
Yes, but not in direct patient care	13% (4)	11% (1)	20% (2)
No	47% (14)	22% (2)	50% (5)
Number of children, % (n)			
1	47% (14)	56% (5)	40% (4)
2	43% (13)	33% (3)	50% (5)
3	10% (3)	11% (1)	10% (1)
Duration of pregnancy, weeks			
Mean (SD)	39.3 (1.4)	39.3 (1.2)	38.9 (1.8)
Median (min, max)	39.5 (33.9, 41.3)	39.1 (37.4, 41.0)	39.4 (33.9, 40.1)
Infant sex, % (n)			
Male	43% (13)	33% (3)	60% (6)
Female	57% (17)	67% (6)	40% (4)
**Primary Maternal COVID-19 Vaccine**	**(n=33)**		
Manufacturer, % (n)			
Pfizer-BioNTech	61% (20)	67% (6)	40% (4)
Moderna	39% (13)	33% (3)	60% (6)
Timing of 1st dose, % (n)			
During pregnancy	76% (25)	78% (7)	90% (9)
During postpartum period	24% (8)	22% (2)	10% (1)

a The sample size for some variables is reduced due to missing survey data from 3 participants. The 3^rd^ dose subgroups (n = 19) did not differ significantly on any variable in this table.

**Table 2:** Symptoms after 3rd vaccine dose

Symptoms	Total 3rd dose sample (n = 26)
**Injection site symptoms, % (n)**	
Any injection site symptoms	73% (19)
Pain	62% (16)
Redness	4% (1)
Swelling	8% (2)
Itching	–
Rash near injection site	–
**General symptoms, % (n)**	
Any general symptoms	65% (17)
Fever	12% (3)
Chills	12% (3)
Headache	27% (7)
Joint pain	12% (3)
Muscle/body aches	31% (8)
Fatigue or tiredness	50% (13)
Lump/swelling in breast (opposite side of injection)	4% (1)

Additional symptoms assessed but not reported by any participant after 3rd dose: nausea vomiting, diarrhea, abdominal pain, rash, mastitis, decreased milk supply or other symptom not listed above.

**Table 3:** Clinical characteristics of participants in the 3rd dose subgroups

Characteristics	3rd dose subgroups	
	Uninfected (n = 9)	COVID-19 infection (n = 10)
**Maternal vaccine-related characteristics**		
Third vaccine dose received, % (n)		
BTN162b2 (Pfizer-BioNTech)	44% (4)	30% (3)
mRNA-1273 (Moderna)	56% (5)	70% (7)
Infant age at 3rd dose, months		
Mean (SD)	6.8 (3.7)	4.7 (3.4)
Median (min, max)	5.5 (0.8, 11.9)	5.2 (−1.7^a^, 9.4)
Days between third dose and symptom assessment		
Mean (SD)	83 (92)	121 (85)
Median (min, max)	42 (15, 260)	91.5 (29, 269)
**SARS-CoV-2 infection characteristics**^b^		
SARS-CoV-2 infections, % (n)		
Mother and infant		80% (8)
Mother only		20% (2)
Neither mother nor infant	100% (9)	
Infant age at time of maternal infection, months		
Mean (SD)		7.1 (3.5)
Median (min, max)		7.8 (−0.8^a^, 12.0)
Days between maternal 3rd dose and infection		
Mean (SD)		72 (38)
Median (min, max)		72 (23, 141)

The 3rd dose subgroups did not differ significantly on any variable in this table (comparisons exclude the 3 participants included in both subgroups).

a One infant had a negative age at both the maternal 3rd dose and maternal infection because both occurred during pregnancy, prior to the infant’s birth.

b Of the 10 COVID-19 infections, 2 were diagnosed in late December 2021, 5 in January 2022, 2 in February 2022, and 1 in early March 2022.

### Symptomatology following 3^rd^ mRNA vaccine dose and/or SARS-CoV-2 infection

**Figure 2:**
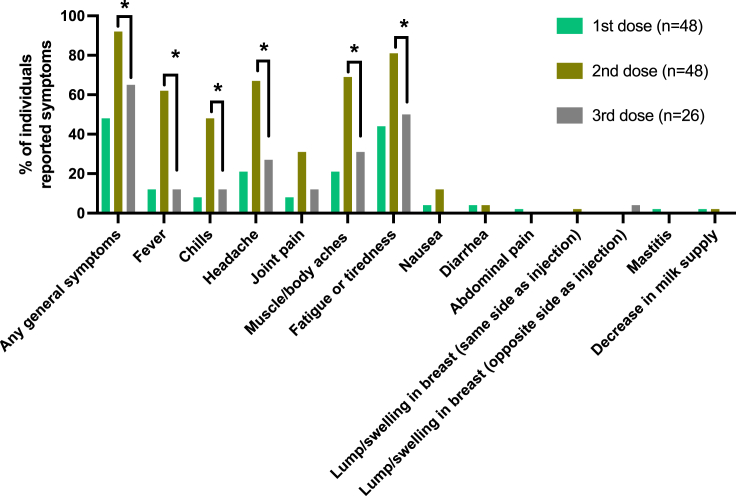
Symptomatology following mRNA vaccine doses Fisher’s Exact test for independent samples was perform to compare symptoms reported after 3^rd^ dose to those reported after 1^st^ and 2^nd^ dose in lactating individuals, as previously reported.^12^ Asterisks indicate symptoms significantly different between 2^nd^ and 3^rd^ dose (p value < 0.01). No significant differences were observed between 1^st^ and 3^rd^ dose. Bars represent value.

Patient reported symptoms were collected by REDCap surveys at least 2 weeks after exposure to a 3^rd^ mRNA vaccine dose and/or infection. No severe symptoms were reported after the 3^rd^ vaccine dose in this cohort (Table 2). The most common maternal symptoms were pain in the injection site, reported by 16/26 participants (62%), or fatigue and tiredness, reported by 13/26 participants (50%). Maternal post 3^rd^ dose symptoms were significantly lower compared to symptoms reported in similar cohort of lactating individuals after 2^nd^ dose,^12^ and were not significantly different from reports after 1^st^ dose (Figure 2). In addition, symptoms reported in our cohort were similar to rates reported in larger cohorts.^25^ When comparing post 3^rd^ dose and post-infection symptoms of the individuals with infection in this cohort (n=10) we found that general symptoms were more likely to be reported by these participants after SARS-CoV-2 infection than after the 3^rd^ dose (p=0.025 for McNemar’s chi-square test) (Table 4). No infant symptoms were reported by mothers after receiving the 3^rd^ dose (n=26); however, all infants that were infected with SARS-CoV-2 at the time of this study (n=8) had at least one symptom reported, including cough, runny nose, and fever (Table 5). No infants were hospitalized after SARS-CoV-2 infection in this cohort, but one infant required evaluation in the Emergency Department for their SARS-CoV-2 infection symptoms. Additionally, in seven of eight infected infants, surveyed mothers reported consultation with their physician about the infant’s SARS-CoV-2 infection (Table 5). The infected infants were on average 8 months old (range 5–12 months) and were not exclusively breastfed at this age (supplemented with formula or with complementary foods).

**Table 4:** Comparison of post 3rd dose symptoms and COVID symptoms for participants with SARS-CoV-2 infection (n = 10 paired analysis)

Symptoms	Timing of symptoms	
	After 3rd dose	During SARS-CoV-2 infection
**General symptoms**		
Any symptoms	50% (5)^a^	100% (10)^a^
Fever	20 (2)	30% (3)
Chills	10% (1)	20% (2)
Headache	10% (1)	50% (5)
Muscle/body aches	20% (2)	40% (4)
Fatigue or tiredness	40% (4)	50% (5)
Nausea	–	10% (1)
**COVID-specific symptoms**		
Sore throat	b	40% (4)
Shortness of breath	b	–
Cough	b	60% (6)
Loss of taste or smell	b	10% (1)
Runny or congested nose	b	80% (8)
Eye redness or discharge	b	–

In this small sample, individual symptom frequencies after 3rd dose and during SARS-CoV-2 Omicron infection were not significantly different. Additional symptoms assessed but not reported by any participant either after 3rd dose or during SARS-CoV-2 infection: vomiting, diarrhea, abdominal pain, rash, lump/swelling in breast, mastitis, decreased milk supply or other symptom not listed above.

a Symptoms were more likely to be reported during Omicron infection than after the 3rd vaccine dose (p = .025 for McNemar’s chi-square test).

b COVID-specific symptoms were only assessed after Omicron infection, not after 3rd dose.

**Table 5:** Infant symptoms after 3rd maternal vaccine dose and during SARS-CoV-2 infection

**INFANT SYMPTOMS AFTER THIRD MATERNAL VACCINE DOSE**	**% (n)**
None reported among the 26 participants who completed the symptom survey after their third vaccine dose	0% (0)

**INFANT SYMPTOMS DURING SARS-COV-2 INFECTION (n=8)**	**% (n)**

Any symptoms	100% (8)
Cough	88% (7)
Runny nose	88% (7)
Fever	50% (4)
Fatigue	25% (2)
Loss of appetite	38% (3)
Eye redness	12% (1)
Diarrhea	12% (1)
Swollen lymph nodes	12% (1)
Joint pain	12% (1)
Muscle/body aches	12% (1)
Other symptoms:	25% (2)
Mildly fussy/woke up more frequently during the night. Mild fever and fussiness/fatigue lasted ∼24 h but runny nose persisted for a week	
Mild increased work of breathing and mild tachypnea	

**TREATMENT FOR INFANT SARS-CoV-2 INFECTION (n=8)**	
Consulted with pediatrician regarding SARS-CoV-2 infection	88% (7)
Treated with anti-pyretics	12% (1)
Evaluated in Emergency Department for SARS-CoV-2 infection	12% (1)
Infant was hospitalized for SARS-CoV-2 Infection	–

Symptoms asked about, but not reported for any infant: rash, hand or foot swelling, redness of tongue, shortness of breath, chest discomfort/pain, loss of taste or smell, headache, dizziness, vertigo, insomnia, hair loss, persistent sweating, impaired memory, poor concentration.

### Longitudinal persistence of anti-SARS-CoV-2 milk antibodies after vaccination and differential milk IgA responses following SARS-CoV-2 infection compared to post-vaccination

**Figure 3:**
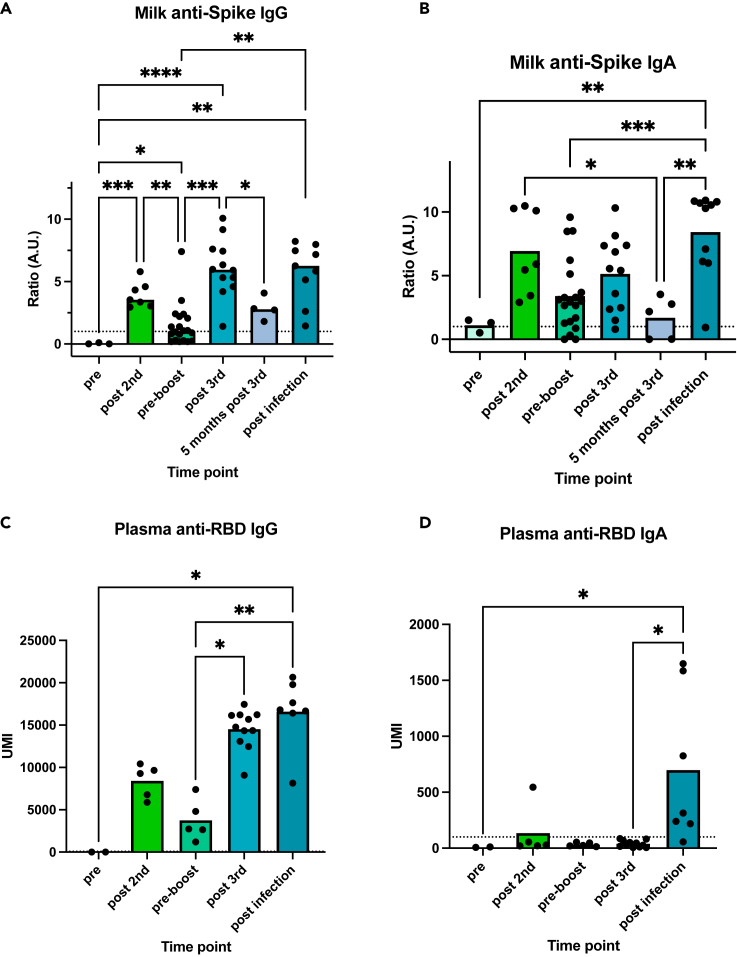
Longitudinal persistence of anti-SARS-CoV-2 milk antibodies after vaccination and differential milk IgA responses following SARS-CoV-2 infection compared to post-vaccination (A–D) Anti-Spike IgG (A) and IgA (B) were measured in human milk samples and anti-RBD IgA (C) and IgG (D) were measured in plasma samples by Luminex assay, at multiple time points as represented in the X axis. Samples collected (1) pre-vaccine (2) post-dose 2 (range 4.7 to 7 weeks following 2^nd^ dose; (3) pre-boost (prior to 3^rd^ dose, range 26–37 weeks following 2^nd^ dose); (4) post-dose 3 (range 4–10 weeks following 3^rd^ dose; (5) post-infection (range 2–7 weeks following infection accruing after 3-dose vaccination series), and (6) 5 months after post-dose 3 (range 18–21 weeks after 3^rd^ dose). Dotted lines indicate the lower cut-off (aforementioned 1 considered positive). Upper limit of detection was at a ratio of 8.5. Asterisks represent p values: ∗= p value < 0.05, ∗∗= p value < 0.01, ∗∗∗= <0.001, ∗∗∗∗= <0.0001 as determined by unpaired Mann-Whitney test. Bars represent mean.

Milk anti-Spike IgG antibodies were detected 6–8 months following the 2^nd^ dose (pre-boost), but significantly decreased over time—with only 52% (10 of 19) of individuals had detectable antibodies in milk prior to 3^rd^ dose boost vaccination (Figure 3A, pre-boost). In contrast to IgG, 16 of 19 (84%) of individuals maintained detectable levels of milk anti-Spike IgA after the 2^nd^ dose, and prior to 3^rd^ dose boosting, but there was also a significant decrease in these antibody levels over time (Figure 3B, pre-boost). After the 3^rd^ dose, milk anti-Spike IgG levels increased significantly, and were significantly higher compared to their levels following the 2^nd^ dose (Figure 3A, post-3^rd^). Milk anti-Spike IgA levels also trended higher after receipt of the 3^rd^ dose but was not statistically significantly increased over pre-boost levels and their levels were similar to the post 2^nd^ dose time point indicating a persistence of anti-Spike milk IgA over time after primary vaccination series, but a lack of significant boosting of milk anti-Spike IgA levels after the 3^rd^ dose. Both IgG and IgA levels decreased 5 months after the 3^rd^ dose, but in contrast to the pre-boost time point all participants had detectable IgG levels and only 3 of 5 (60%) had detectable IgA levels at this time point (Figures 3A and 3B). Individuals with SARS-CoV-2 infection after their 3^rd^ dose had significantly higher levels of IgA in their milk following infection (Figure 3B, post infection) compared to individuals after 2^nd^ and 3^rd^ vaccine doses. However, milk anti-Spike IgG levels after infection were comparable to the levels after 3^rd^ dose (Figure 3A). Similar results were obtained when analyzing plasma antibody levels in a subgroup of participants with blood samples. We found a significant increase in IgG levels after 3^rd^ dose with no further increase after infection (Figure 3C). Furthermore, we found higher levels of anti-Spike IgA antibodies in the plasma of lactating individuals after infection compared to after the 3^rd^ dose (Figure 3D).

### Milk RBD-blocking activity increases after vaccination and infection

**Figure 4:**
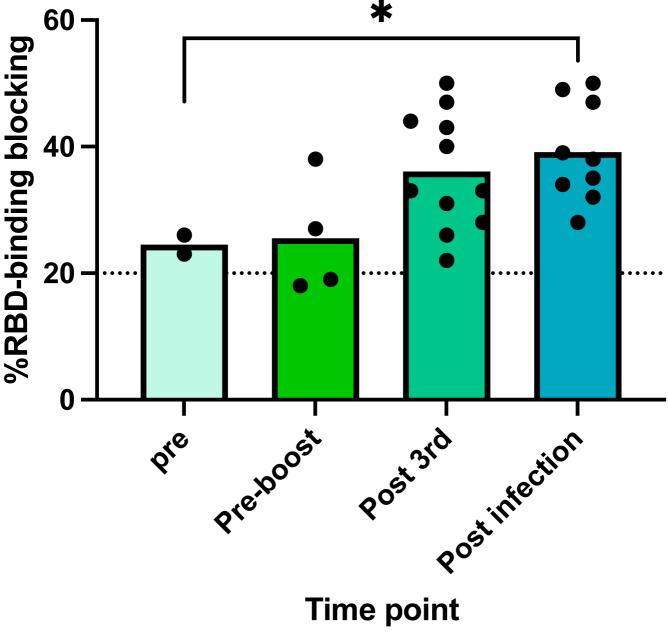
Milk RBD-blocking activity increases after vaccination and infection Milk samples were analyzed for neutralization activity (RBD binding capacity) using ELISA assay. Asterisks represent p values: ∗= p value < 0.05 as determined by unpaired Mann-Whitney test. Bars represent mean.

Milk samples collected prior to any vaccination and before 3^rd^ dose had low-level SARS-CoV-2 blocking activity as measured by RBD-binding assay (mean 24.5% neutralization n=2, mean 25.5% neutralization n=4). After 3^rd^ dose, there was a trend of an increase in RBD-binding blocking activity, however, it was not significantly different from the pre-vaccine and pre-3^rd^ dose samples (mean 36% neutralization n=10 p=0.11). We then evaluated post-infection milk samples and found significantly higher RBD-binding blocking activity compared to the pre-vaccine milk samples (mean 39% neutralization n=9 p=0.03) (Figure 4).

### Persistence of maternal milk-derived SARS-CoV-2 antibodies in infant saliva after breastfeeding

**Figure 5:**
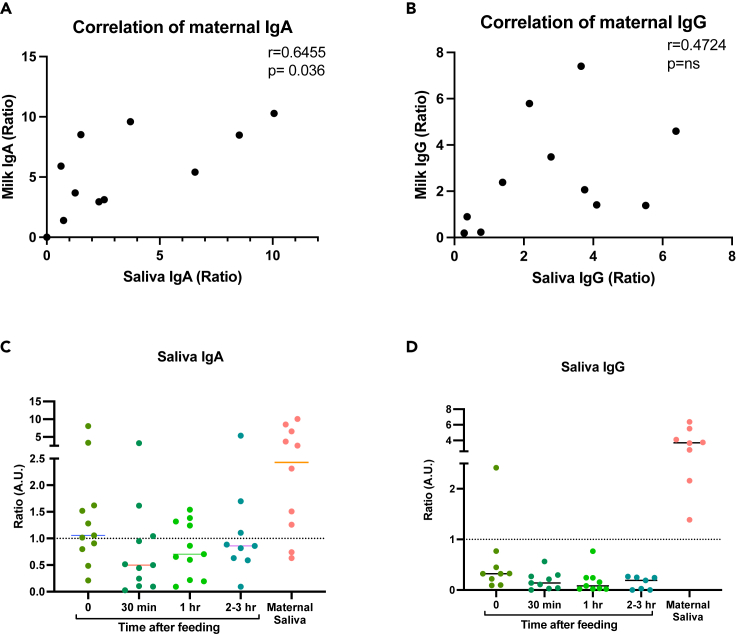
Persistence of maternal milk-derived SARS-CoV-2 antibodies in infant saliva after breastfeeding Milk and saliva samples were collected from mother and infants that were vaccinated with 2 doses of mRNA-based vaccine. (A–D) Two-tailed Spearman correlation was used to correlate milk and maternal saliva anti-Spike IgA levels (A) and IgG (B). In addition, anti-Spike IgA levels (C) and IgG (D) infant saliva samples were measured at multiple time points, immediately after breastfeeding (0), 30 min (30 min) after feeding, 1 h after feeding (1 h) and before next feeding (2–3 h after feeding). Maternal saliva was collected at the same day for comparison. Dotted lines indicate the lower cut-off (aforementioned 1 considered positive). Lines represent mean.

Milk antibodies may provide protection to the infant at the oropharyngeal mucosal surfaces, but little is known regarding the stability of these antibodies in the infant mouth after breastfeeding. To answer this question, we investigated the stability and persistence of milk antibodies in infant saliva after breastfeeding using saliva samples collected from infants at multiple time points after feeding. We compared these to antibody levels in maternal milk and saliva samples collected the same day as the infant. We found a positive correlation between anti-Spike IgA levels in milk and maternal saliva samples (Figure 5A), as well as a positive but non-significant correlation for milk and maternal saliva anti-Spike IgG antibodies (Figure 5B). We next evaluated infant saliva samples collected after feeding by mothers who had detectable anti-Spike IgG or IgA in their milk. Anti-Spike IgA levels were found to be significantly higher in infant saliva over time after feeding compared to IgG antibodies, with 6/11 (55%) infants having detectable antibodies immediately after breastfeeding and 3/11 (27%) infants remaining positive at all time points until the next feeding. We found that IgG antibodies were less abundant in the infant’s saliva after feeding, with all except one infant’s samples below the assay cut-off (Figures 5C and 5D). Of note, we could not identify any correlation between infant age and lactation exclusivity to infant saliva antibodies, which may be due to the small sample size of this study.

## Discussion

We found that mRNA-based vaccines administered in pregnancy or during lactation stimulated increased anti-SARS-CoV-2 spike antibody levels in human milk that persisted for up to 8 months after vaccination. In addition, we found that a 3^rd^ dose (booster) significantly increased the IgG antibody levels in milk, and to a lesser extent IgA, however, IgA was more persistent over time following primary vaccination series. Additionally, we found a significant boosting of IgA levels after SARS-CoV-2 infection, in both maternal plasma and milk. Lastly, we found the presence of transferred human milk SARS-CoV-2 IgA antibody in infant saliva that persisted following breastfeeding. Taken together, vaccination during lactation imparts additional transfer of antibodies through human milk, which may provide further protection against SARS-CoV-2 to young infants, who are currently not eligible for COVID-19 vaccination until they reach 6 months of age.

We performed a comparative analysis of anti-Spike milk IgG and IgA levels that are induced after 2 doses vs. 3 doses of the mRNA vaccine, as well as SARS-CoV-2 infection after 3^rd^ dose. Although anti-SARS-CoV-2 IgA antibodies are induced after vaccination, their levels are significantly boosted after natural infection as compared to vaccination alone. In contrast, milk anti-SARS-CoV-2 IgG levels did not significantly increase after infection. Of note, milk IgG levels were very high after the 3^rd^ dose and may have already reached peak levels at the time of infections, which occurred within 7 weeks of the 3^rd^ dose in our cohort. Previous studies have also shown similar patterns of IgA in milk and saliva,^26^^,^^27^ indicating that exposure to SARS-CoV-2 infection has a greater effect on mucosal IgA secretion compared to vaccination. Interestingly, mucosal and systemically delivered influenza vaccines result in similar increases in influenza specific IgA antibodies in milk^28^ suggesting that factors in addition to exposure location may affect the production of milk IgA antibodies.

IgA antibodies play a critical role in humoral immune response and virus neutralization, with peripheral expansion of IgA plasmablasts found in SARS-CoV-2 infected patients shortly after the onset of symptoms.^29^ Previous studies have shown that mucosal immunity in the bronchoalveolar lavage fluid is weaker after vaccination compared to post-infection immunity.^30^ In addition, SARS-CoV-2 infection prior to vaccination was shown to induce a better secretion of antigen-specific mucosal secretory IgA to the saliva, compared to vaccination alone.^31^ Our findings further suggest that mRNA-vaccines induced an IgA response in milk and in blood, but to a lower extent compared to hybrid immunity from vaccination and SARS-CoV-2 infection. To the best of our knowledge, our work is the first to compare boosting of milk antibody levels after the 3rd dose versus infection during the time that the Omicron variant was the predominant circulating strain.

We also performed an RBD-blocking activity assay on milk samples and found higher but not significantly increased RBD-blocking activity after vaccination, but did find significantly elevated RBD-blocking activity in samples collected after infection. This assay is not variant specific, but examines the total RBD-binding activity that blocks the virus from binding to the ACE2 receptor. Although we tested a small number of samples, we found that pre-vaccine samples have some baseline RBD-blocking activity likely due to other milk components with antiviral activity such as lactoferrin and MUC1 that are highly expressed in human milk.^32^^,^^33^^,^^34^ In addition, we did see a significant increase in RBD-blocking activity after infection, suggesting again that milk antibodies have the capacity to neutralize the SARS-CoV-2 virus.^14^^,^^21^^,^^35^^,^^36^

In addition, to our knowledge our paper is the first to measure the persistence of anti-SARS-CoV-2 antibodies in infant saliva in multiple time points after feeding after maternal vaccination. A previous study measuring anti-SARS-CoV-2 antibodies in saliva of infants born to infected mothers suggests that breastfed infants have higher levels of these antibodies compared to formula fed infants.^37^ They also suggested that those antibodies are produced by the infants after exposing to immune complexes from milk.^37^ We found that SARS-CoV-2 IgA antibodies are more abundant in infant saliva at multiple time points after breastfeeding compared to IgG. Others have shown that SARS-CoV-2 IgG antibodies are present in infant’s stool samples,^10^ suggesting IgG may transit quickly through the infant’s oropharynx, and may play a role in other mucosal organs such as the lower gastrointestinal tract. Therefore, developing vaccines that improve the secretion of IgA antibodies to milk (and other mucosal organs) might also better contribute to infant (and maternal) protection against infection, particularly respiratory-transmitted pathogens. Larger studies are needed to evaluate the protective effects of anti-SARS-CoV-2 milk-derived antibodies on breastfed infants.

Eight infants in our cohort were infected with SARS-CoV-2 during the study period (when the Omicron variant was predominant^38^), in the setting of maternal infection, despite their mother having received the 3^rd^ vaccine dose. Regardless of the persistence of anti-SARS-CoV-2 antibodies in milk over time, passively derived milk antibodies alone were insufficient to fully protect against infection, possibly due to immune evasion from vaccine-induced antibodies by the Omicron variant, and/or weaker protection provided by milk antibodies compared to passively acquired transplacentally transferred IgG systemic antibodies that wane after birth.^39^^,^^40^ All infants infected in this cohort were older than 5 months, so their transplacental antibodies were lower or absent at time of infection, and most of them were not exclusively breastfed when infected (were supplemented with baby formula or complementary foods). However, due to our limited sample size, we were unable to assess the level of protection provided by transfer of vaccine-related antibodies in human milk, as compared to infants with no SARS-CoV-2 vaccine-related milk antibodies. The Center for Disease Control and Prevention (CDC) reported that during the early-2022 Omicron variant peak, infants hospitalization rates were 5 times higher compared to during the Delta variant peak.^22^ All SARS-CoV-2 infected infants in this cohort presented with symptoms, and one infant was admitted for evaluation in emergency care unit for COVID-19 symptoms. These results underscore the importance of both passive maternally derived and early infancy vaccination protection for this vulnerable infant population. In contrast to infants infected with SARS-CoV-2, no infants in our cohort were reported to have symptoms following maternal COVID-19 vaccination during lactation. In a larger cohort that examined 10,278 participants after 3rd dose, 1.2% of mothers reported any issue in their infant after vaccination during lactation.^25^ These reports emphasize the importance of including lactating individuals in clinical trials, to be able to examine the direct effect of vaccine administration on infant symptoms, in compared to placebo group, which are currently absent.

In summary, we found that human milk antibody levels increase significantly after the 2^nd^ vaccine dose and can maintain high levels in milk up to 8 months post vaccination in some individuals. Boosting with a 3^rd^ vaccine dose significantly increases IgG antibody levels that remain elevated for at least an additional 5 months post-booster vaccination in milk. Milk IgA antibodies were much more significantly increased after SARS-CoV-2 infection, compared to vaccination alone. Based on our results, it is notable that IgA antibodies, compared to IgG, were more stable in the infant mouth after feeding—and may be more important in infant protection against SARS-CoV-2 infection. Further large-scale cohort studies of vaccinated lactating individuals are needed to better understand the role of milk antibodies in infant protection from SARS-CoV-2 infection. Future vaccine development should focus on the induction of milk IgA antibodies to enhance infant protection during lactation.

### Limitations of the study

This study follow up on a small cohort of participants from the COVIPAL (COVID-19 Vaccine in Pregnancy and Lactation) study, that received 3^rd^ vaccine dose and were infected during the Omicron wave (SARS-CoV-2 B.1.1.529) in the San Francisco Bay Area. Further large-scale studies, in diverse populations should be conducted in lactating participants with varied SARS-CoV-2 variant infection histories and non-mRNA vaccination administration, both nationally and globally, to further strength our findings. The infant saliva sub-study presented here is unique due to the multiple time points collection after feeding, however, larger studies are needed to further address the persistence of milk antibodies in infants’ mucosal organs and should also include biospecimen collection of saliva, nasal swabs, and stool samples from breastfeeding infants in multiple time points after maternal vaccination and infection.

## STAR★Methods

### Key resources table

**Table:** 

REAGENT or RESOURCE	SOURCE	IDENTIFIER
**Critical commercial assays**		

Anti-Spike ELISA assay	Euroimmune, Germany	EI 2606-9601 A
Anti-Spike ELISA assay	Euroimmune, Germany	EI 2606-9601 G
Amicon Ultra Centrifugal Filters	Millipore Sigma, USA	UFC5199BK
SARS-CoV-2 Neutralizing Ab ELISA Kit	Invitrogen	BMS2326

### Resource availability

#### Lead contact

Further information and requests for resources and reagents should be directed to and will be fulfilled by the lead contact, Dr. Mary Prahl (mary.prahl@ucsf.edu).

#### Materials availability

This study did not generate new unique reagents.

### Experimental model and study participant details

#### Participant cohort and data collection

The institutional review board of the University of California, San Francisco, approved the study (#21-33621). Written informed consent was obtained from all study volunteers as part of the COVID-19 Vaccine in Pregnancy and Lactation (COVIPAL) cohort study. Pregnant or lactating mRNA COVID-19 vaccination recipients were enrolled from December 2020 to April 2022. All participants were female, and the mean age of the cohort was 37.2 years (Table 1). Race/Ethnicity was collected by self-reported REDCap questionnaire as follows: 20% identified as Asian, 3% Black or African American, 3% Hispanic/Latina, 70% White/Caucasian, 3% More than 1 race/ethnicity (Table 1). Clinical data and symptoms were collected by medical record review and REDCap questionnaires and characterized in Table 1. Participants were surveyed following each COVID-19 vaccine dose, which included questions about maternal and infant symptomology after maternal 3^rd^ dose. In February 2022 all COVIPAL participants were surveyed if they had a new diagnosis of SARS-CoV-2 infection since the last vaccine dose and if the participant was willing to provide post-infection biospecimen samples. Individuals with SARS-CoV-2 infection (confirmed by PCR or rapid antigen testing) were administered questionnaires to capture maternal and infant post infection symptoms. For individuals at the time of post infection survey that had not yet completed their 3^rd^ dose questionnaire, their 3^rd^ dose symptoms were captured at the same time of their post infection symptoms (up to 6 months after receiving the 3^rd^ vaccine dose). No infants were vaccinated during the study period.

#### Milk sample collection and processing

Milk samples were collected at the following time points: 1) Pre-vaccine 2) Post-Dose 2 (range 4.7 to 7 weeks following 2^nd^ dose; 3) Pre-Boost (prior to 3^rd^ dose, range 26-37 weeks following 2^nd^ dose); 4) Post-Dose 3 (range 4-10 weeks following 3^rd^ dose; 5) 5 months after Post-Dose 3 (range 18-21 weeks after 3^rd^ dose) and 6) Post-Infection (range 2-7 weeks following infection accruing after 3-dose vaccination series) (Figure 1). Fresh human milk samples were self-collected by participants into sterile containers. Milk samples were either processed immediately by the study staff or frozen by mothers in their home freezer as soon as possible after pumping. Samples were transported on ice from participant’s home to the lab for processing. Milk was aliquoted and stored at -80°C until analyzed.

#### Blood sample collection and processing

Paired maternal blood samples were collected at the same time points as described above for milk samples from a subset of participants (n=18). Whole blood was collected into EDTA tubes. Plasma was isolated from whole blood by centrifugation and immediately cryopreserved at -80°C until analysis. Plasma samples were diluted at 1:1000 for IgG detection, and 1:100 for IgA detection.

#### Infant and maternal saliva sample collection

To evaluate the duration of persistence of antibodies in the infant’s mouth after breastfeeding, we collected saliva samples from breastfeeding infants at the following timepoints: 1) immediately after breastfeeding 2) 30 min after breastfeeding 3) 60 min after breastfeeding and 4) before next breastfeeding (2-3 hours after feeding). Paired maternal saliva and milk samples were collected from the infant’s mother on the day of infant collection (n=11). Maternal saliva samples were collected with OraSure collection kits by placing the swab in the mouth for 5 minutes until saturated. For infant saliva samples, sponges for assisted saliva collection were used and were inserted to OraSure collection tubes (Product CS-2, DNAGenotek Inc, Ontario, Canada).

### Method details

#### ELISA assay

Anti-Spike ELISA assay (Euroimmune, Germany) was used to measure IgA and/or IgG levels in blood, skim milk, and saliva samples. Plasma and skim milk samples were thawed on ice. After thawing, milk fat was separated by cold centrifugation (10,000g for 10 min, 4°C) and diluted 1:4 with the provided diluent buffer, and examined using the manufacturer’s protocol as described, with an additional blocking step with 5% BSA in TBS with 0.5% Tween 20 for 30 min before loading the samples as recommended to increase specificity.^41^ Plasma samples were diluted 1:101 and were examined by the same protocol as milk samples. OD values of samples were calculated by dividing by the calibrator OD value (provided with the kit); values with sample:calibrator ratio greater than 1 were considered positive. Saliva samples were centrifuged at 15,000 rpm for 10 min (4°C), and 300ul were transferred to Amicon Ultra Centrifugal Filters (UFC5199BK, Millipore Sigma, USA) and centrifuged at 4°C, 15,000 rpm for 8 min. The concentrated sample was then recovered via a second centrifugation step (4°C, 15,000 rpm for 5 min); this procedure produced ∼ 50 μl of concentrated sample. 50 μl of a blocking buffer prepared in house (5% BSA in TBS with 0.5% Tween 20) was then added before the total volume (∼ 100 μl) was placed in each well of the ELISA plate, and the protocol run as described above. Samples were analyzed in duplicate; and saliva samples were analyzed once for each antibody (IgA and IgG) due to limited sample availability.

#### Milk RBD blocking capacity ELISA assay

Milk samples were analyzed for milk antibody neutralization by binding to the RBD subunit of the SARS-CoV-2 virus using SARS-CoV-2 Neutralizing Ab ELISA Kit (Invitrogen). Milk supernatant samples were diluted 1:32 and were run on the ELISA assay as recommended by the manufacturer and ran in parallel to kit positive and negative controls. Per manufacturer’s recommendations, responses were considered positive if ≥ 20% neutralization and negative if <20% neutralization. Although RBD is the main binding site for SARS-CoV-2, it is important to note that this assay is optimized for plasma and serum. In addition to antibodies, human milk contains a number of other components that may neutralize and/or limit viral replication.^42^

#### Multiplex bead-based assay of SARS-CoV-2 specific binding antibodies-

IgG and IgA antibodies against the receptor binding domain (RBD) of the SARS-CoV-2 virus were analyzed with a multiplex-based human serology kit (Bio-rad, CA, USA) according to the manufacturer’s instructions. Briefly, diluted samples were incubated with coupled beads for 30 minutes at room temperature (RT), followed by incubation with secondary antibodies and streptavidin-phycoerythrin. After proper wash and resuspension of the beads, the reactions were read on a BioPlex-200 equipment (Bio-Rad), and the results were expressed as median fluorescence intensity (MFI).

### Quantification and statistical analysis

Spearman analysis was used for correlation between milk and maternal saliva antibody levels. Welch ANOVA multiple comparison test was used to evaluate differences between the different time points in milk and plasma antibody levels. Wilcoxon matched-pairs signed rank test was used to compared IgA and IgG levels in infant saliva at the different time points. Comparison of symptoms reported by each participant after 3^rd^ dose and after infection was performed by McNemar’s chi-square test. Fisher’s Exact test was used to compared post vaccine symptoms after 1, 2 or 3 doses. Data about 1^st^ and 2^nd^ dose were previously reported.^12^ Statistical analysis was performed using Prism version 9.1.0 (GraphPad) and Stata Version 15.0 were used for analyses. Asterisks represent p-values: ∗= p-value <0.05, ∗∗= p-value <0.01, ∗∗∗= <0.001, ∗∗∗∗= <0.0001.

## Data Availability

All data reported in this paper will be shared by the lead contact upon request. This paper does not report original code. Any additional information required to reanalyze the data reported in this paper is available from the lead contact upon request.
